# Provocation of Symmetry/Ordering Symptoms in *Anorexia nervosa*: A Functional Neuroimaging Study

**DOI:** 10.1371/journal.pone.0097998

**Published:** 2014-05-20

**Authors:** Masashi Suda, Samantha J. Brooks, Vincent Giampietro, Rudolf Uher, David Mataix-Cols, Michael J. Brammer, Steven C. R. Williams, Janet Treasure, Iain C. Campbell

**Affiliations:** 1 Section of Eating Disorders, Institute of Psychiatry, King's College London, London, United Kingdom; 2 Department of Neuroscience, Uppsala University, Uppsala, Sweden; 3 Centre for Neuroimaging Studies, Institute of Psychiatry, King's College London, London, United Kingdom; 4 Departments of Psychosis Studies and Psychology, Institute of Psychiatry, King's College London, London, United Kingdom; University of Udine, Italy

## Abstract

Anorexia nervosa (AN), obsessive–compulsive disorder (OCD), and obsessive–compulsive personality disorder (OCPD) are often co-morbid; however, the aetiology of such co-morbidity has not been well investigated. This study examined brain activation in women with AN and in healthy control (HC) women during the provocation of symmetry/ordering-related anxiety. During provocation, patients with AN showed more anxiety compared to HCs, which was correlated with the severity of symmetry/ordering symptoms. Activation in the right parietal lobe and right prefrontal cortex (rPFC) in response to provocation was reduced in the AN group compared with the HC group. The reduced right parietal activation observed in the AN group is consistent with parietal lobe involvement in visuospatial cognition and with studies of OCD reporting an association between structural abnormalities in this region and the severity of ‘ordering’ symptoms. Reduced rPFC activation in response to symmetry/ordering provocation has similarities with some, but not all, data collected from patients with AN who were exposed to images of food and bodies. Furthermore, the combination of data from the AN and HC groups showed that rPFC activation during symptom provocation was inversely correlated with the severity of symmetry/ordering symptoms. These data suggest that individuals with AN have a diminished ability to cognitively deal with illness-associated symptoms of provocation. Furthermore, our data also suggest that symptom provocation can progressively overload attempts by the rPFC to exert cognitive control. These findings are discussed in the context of the current neurobiological models of AN.

## Introduction

The aetiology of anorexia nervosa (AN) is unclear [1], and the treatment of this disorder requires vast improvement [2], [3]. Hence, there is a need to increase our understanding of the neurobiology of this condition [3] and how it relates to other eating disorders (ED), as well as to conditions that are often co-morbid with AN as these may be both causal and maintenance factors in this disease [4]. Anxiety disorders, particularly social anxiety and obsessive–compulsive disorder (OCD), are commonly co-morbid with and often precede the onset of AN. AN has a co-morbidity rate of 20–40% for OCD and a 20–30% co-morbidity rate for obsessive–compulsive personality disorder (OCPD) [5]. Moreover, girls with OCD have a higher risk of developing an ED later in life [6]. The type of OCD behaviours that often present in patients with AN have been described as a need for symmetry or exactness, rather than aggressive obsessions and checking compulsions [7]. Moreover, this need for symmetry remains after long-term recovery from AN [8]. In addition, one family study reported that the risk of OCPD was elevated only among relatives of anorexic probands, indicating these two disorders may involve shared familial risk factors [9]. Therefore, these results suggest a shared familial transmission of AN and OCPD, which raises the possibility that it is necessary to have a risk for both OCPD and an ED in order to develop AN. Thus, OCPD traits, such as the need for symmetry and exactness, are not merely correlates of AN, but may also be intermediate phenotypes.

Current neurobiological models propose that AN involves anomalies in the corticolimbic pathways (lateral amygdala, medial prefrontal cortex, and orbitofrontal cortex) associated with anxiety, hypervigilance, and affective instability [10], as well as differences in the functioning of the frontostriatal pathways (lateral striatum, medial prefrontal cortex, and orbitofrontal cortex) associated with compulsivity, motivation, and habits [11]. These brain regions provide targets for emerging neural-based treatments such as repetitive transcranial magnetic stimulation (rTMS) and deep brain stimulation (DBS) [12].

Functional magnetic resonance imaging (fMRI) studies of AN have generally focused on specific aspects underlying the psychopathology of the disorder. For example, many studies have focused on examining brain responses to salient visual cues, such as food images [11], [13]–[17], body images [18]–[25], and/or body checking [26]. Other studies have focused on broader processes that are shared by other forms of psychopathology, such as reward sensitivity [27]–[29], cognitive flexibility [30], social cognition [31], [32], and working memory [33]. Recently, studies have focused on characteristics of the functional connectivity of brain networks [34]–[36] and white matter abnormalities using diffusion tensor imaging techniques [37]. However, to our knowledge, the visual cues that are salient to some features of OCD and anxiety which often present in AN have not yet been extensively studied. These visual cues include, for example, the need for symmetry and exactness. One study used such cues in children with OCD and reported that they elicited higher subjective anxiety, which was associated with reduced activation in the right thalamus and right insula [38]. In addition, using similar cues, a positron emission tomography study of Tourette's syndrome, which comprises symptoms of OCD, reported increased regional cerebral blood flow (rCBF) in the anterior cingulate cortex, supplementary motor area, and inferior frontal cortex [39].

To our knowledge, this is the first neuroimaging study to focus on OCD/OCPD traits in AN. In this fMRI study, the effects of viewing images that provoke concern for symmetry and exactness in individuals with AN were examined in an exploratory manner. In order to assess the possibility raised by Lilenfeld et al. 1998, that OCPD and AN represent a continuum of phenotypic expressions of a similar genotype, we hypothesised that brain activation would be provoked by stimuli associated with symmetry and exactness in patients with AN and that similar patterns of brain activation would be observed in imaging studies of OCD.

## Materials and Methods

### Participants

Forty-six right-handed women aged 17–34 years were enrolled in this study; of these, 22 had a DSM diagnosis of AN and 24 were age-matched healthy controls (HCs) who were in the normal weight range and were without a lifetime diagnosis of a psychiatric disorder. The AN group were recruited from the South London and Maudsley NHS Trust, and the HC group included students (recruited via local advertisements). Diagnoses were made using the Structured Clinical Interview for DSM-IV (SCID) [40]. General exclusion criteria were a history of head injury, hearing or visual impairments, neurological disease, metal implants, and claustrophobia. Written informed consent was obtained from all the participants prior to their inclusion in the study and this approach was approved by the South London and Maudsley NHS Trust Ethics Committee. For minors involved in the study written informed was obtained from the concerned participant and this was approved by ethics committee. Study was approved by the South London and Maudsley NHS Trust Ethics Committee.

### Procedure

Participants were asked to avoid consuming alcohol for 24 h preceding the study onset, and were also instructed to refrain from eating food and drinking caffeinated beverages for 2 h prior to the experiment. Before entering the study, all participants were interviewed (SCID) to screen for psychiatric diseases [40], and completed a package of self-report questionnaires. The Eating Disorder Examination Questionnaire (EDE-Q) assesses behavioural and attitudinal eating pathology [41]. The Hospital Anxiety and Depression Scale (HADS) is a self-report instrument that measures depression and anxiety [42]. OCD symptoms were assessed using the Obsessive–Compulsive Inventory, Revised (OCI-R), which is a self-administered instrument with six dimensions: washing, checking, hoarding, ordering, obsessing, and neutralising [43]. The OCI-R is an 18-item self-reported measure that uses a five-point scale for scoring on six subscales (washing, checking, ordering, obsessing, hoarding, and neutralizing).

### Stimuli

**Figure 1:**
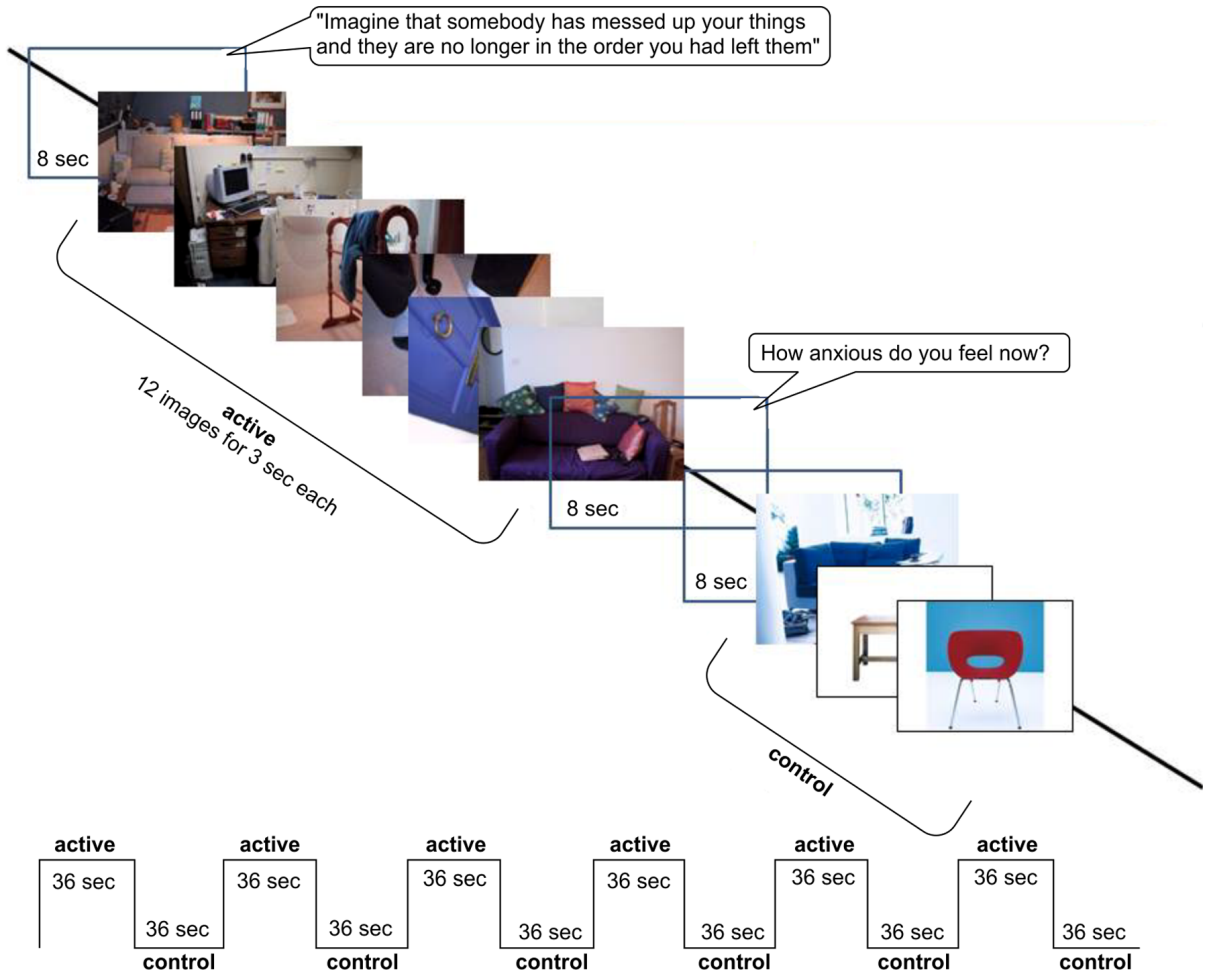
Illustration of the block-design paradigm.

Fifty colour photographs of symmetry/order were randomly presented on a blue background. The selection and evaluation of these images has been previously described [44]. Images were presented on a rear-projection screen and viewed via a double-mirror periscope fitted to the scanner head coil. Twelve symmetry/order images, e.g. uneven/chaotic/messy environments (active condition) were followed by 12 neutral images, e.g. furniture, nature scenes, and household items (control condition). This sequence was repeated six times, and images were presented for 3 s without pause. Each block was preceded by the following audio instruction: ‘imagine that somebody has messed up your things and they are no longer in the order you had left them’. At the end of each block, participants rated their anxiety verbally on a scale from 0 (no anxiety) to 10 (high anxiety) (Figure 1).

### Image acquisition

Functional MRI was performed on a GE Signa HDx 1.5 T scanner (GE Medical Systems, Milwaukee, Wisconsin). T2*-weighted images depicting blood oxygen level-dependent (BOLD) contrasts were acquired every 4 s (repetition time [TR]) with an in-plane voxel size of 3.3×3.3 mm. The echo time was 40 ms, and the flip angle was 90°. Whole-brain coverage was acquired with 43 slices (slice thickness, 3 mm; interslice gap, 0.3 mm) and the matrix size was 64×64 voxels. Fifty-four T2*-weighted whole-brain volumes were acquired in each condition.

### Data analyses

Imaging data were analysed with the XBAM (version 4.1) software, which uses a non-parametric approach for fMRI analysis [45], [46]. Use of a non-parametric analysis was reinforced by a study reporting widespread departure from normality in group fMRI data [47]. After motion correction, spin-excitation history correction, and linear detrending, the estimated BOLD effect was modelled by two gamma variate functions with haemodynamic delays of 4 and 8 s. All subjects were within acceptable limits for head-movement parameters (<3.0 mm); *t*-tests showed no significant group differences in the extent of 3-dimensional motion in x, y, and z translation and rotation. The least-squares model of the weighted sum of the two gamma variate functions was then compared with the signal in each voxel to obtain a goodness-of-fit statistic. The distribution of this statistic under the null hypothesis was calculated by wavelet-based resampling of the time series and refitting the models to the resampled data. Generic group activation maps (active vs. control condition) were constructed by mapping the observed and randomised test statistics into standard space and by computing and testing median activation maps.

Median statistics minimised the impact of outlier effects. Between-group differences were established by a cluster-level analysis with data randomisation between groups to determine the sampling distribution of group differences under the null hypothesis. Group membership was permuted 1000 times, and a null distribution was formed at each voxel; this distribution contained only the randomised voxel statistics at that voxel. The significance of each voxel was assessed against its own null distribution. An identical permutation strategy was applied at all voxels; thus, we were able to subsequently form clusters of spatially contiguous significant voxels. Detection of activated voxels was extended from voxel to cluster level, as described by Bullmore et al. [46]. The probability of the occurrence of any cluster in the observed data was computed by reference to the null distribution (generated from the random clusters occurring in the permuted data). Similar to our previous studies, we set the voxel-wise significance level at *P*≤0.01 [17], [19], while the cluster-wise significance threshold was set to obtain less than one false-positive 3D cluster/map (*P*≤0.001 for group comparisons and *P*≤0.002 for group analyses). At these levels, the cumulative number of expected false positive clusters was <1. In large connected clusters, local maxima that were further apart than the upper bound of the likely Talairach mapping error (3 voxel radius of 10 mm) [47] were identified. Voxels were then assigned to the nearest local maximum with a statistical value exceeding that of the assigned voxel.

To assess the potential effects of clinical variables on between-group differences in neural activity, we extracted mean BOLD signal changes in neural regions that exhibited significantly reduced activation in the AN than in the HC group. Using the Statistical Package for the Social Sciences (SPSS; SPSS Inc., Chicago, IL), we performed stepwise regression analyses, treating clinical variables as independent variables and the extracted mean signal change as dependent variables in the AN and HC groups. We also performed the same regression analysis in the whole group (AN+HC), to avoid the problem of low statistical power resulting from the small sample size of individual groups. We included age, duration of ED, anxiety rating for the task, six predictor variables related to OCD symptoms (checking, hoarding, neutralising, obsessing, ordering, washing), and four predictor variables related to ED symptoms (restraint, eating concern, shape concern, and weight concern) as independent variables.

Within- and between-subject differences in the self-report data were analysed using Student's *t*-tests. Associations between continuous variables were evaluated by Spearman's (rho) correlation coefficient, if appropriate. For group comparisons of subjective anxiety ratings of stimulus images, OCI-R scores, EDE-Q scores, and HADS scores, a Bonferroni adjusted *P*<0.004 was considered significant. In all other cases, *P*<0.05 was considered significant. To compare behavioural results provoked by symmetry/ordering tasks in AN to findings of a previous study on OCD, the mean difference in anxiety ratings for stimulus images between the AN and HC groups was standardised by calculating Cohen's d, which is the difference between the two raw means divided by the pooled standard deviation [48].

## Results

### Participant characteristics

All fMRI scans were conducted between 1∶30 and 4 pm. Two patients with AN were excluded from this study because of motion artefacts during fMRI scanning, therefore, a total of 20 patients with AN were included in the data analyses. Of the 20 patients with AN, 13 were classified as suffering from the restricting form of AN (ANR) while seven patients were categorised with the binge–purge (ANBP) subtype. Findings from the SCID interview yielded the following psychiatric current comorbidity data: six patients had generalised anxiety disorder, one had major depression, one had posttraumatic stress disorder, and four had obsessive compulsive disorder. Seventeen patients were on antidepressant medication, and three were drug naive. As shown in Table 1, the AN group had significantly lower BMIs when compared to the HC group (mean, 15.3 vs. 21.6). The patients' mean age was in the mid-20 s (i.e. similar to that of the HC group), and the average duration of illness in the AN group was 10 years.

**Table 1:** Demographic and psychometric data.

	AN (*n* = 20)	HC (*n* = 24)	AN vs. HC (*P*)
Age	26.8 (8.0)	24.3 (8.0)	0.27
BMI	15.3 (1.1)	21.6 (2.2)	<0.001
Duration of ED	10.0 (7.0)	N/A	
**EDEQ**			
Total score			
Restrained eating	2.8 (1.7)	1.0 (1.2)	<0.001
Eating concern	3.3 (1.4)	0.4 (0.7)	<0.001
Weight concern	3.8 (1.6)	1.0 (1.1)	<0.001
Shape concern	4.6 (1.2)	1.5 (1.4)	<0.001
HADS:	13.4 (3.7)	4.5 (2.5)	<0.001
**Anxiety ratings post-image (0–10)**			
Symmetry images	6.2 (2.3)	3.9 (2.3)	0.003
Control images	1.2 (1.0)	1.1 (0.9)	0.85
**OCI-R**			
Checking	4.6 (3.5)	1.6 (2.5)	0.003
Hoarding	4.0 (2.4)	2.4 (2.6)	0.04
Neutralising	3.4 (3.6)	0.5 (0.9)	<0.001
Obsessing	7.7 (3.5)	1.5 (2.3)	<0.001
Ordering	7.1 (3.3)	2.3 (2.3)	<0.001
Washing	4.1 (4.2)	0.7 (1.1)	<0.001

Values are mean and (SD).

Abbreviations: AN, anorexia nervosa; BN, bulimia nervosa; HC, healthy control; BMI, body mass index; ED, eating disorder; EDEQ, Eating Disorders Examination Questionnaire; HADS, The Hospital Anxiety and Depression Scale; OCI-R, Obsessive Compulsive Inventory Revised.

### EDEQ, HADS, and OCI-R scores

Table 1 shows that the AN group had significantly higher scores on the EDEQ and all of its sub-scales. Individuals in that group also scored higher on the HADS (mean, 13.4 vs. 4.5) and on all of the sub-scales of the OCI-R. In addition, the differences were greatly significant for all sub-scales (*P*<0.005), with the exception of hoarding.

### Subjective anxiety ratings associated with viewing symmetry and control images

Subjective anxiety ratings for symmetry/order images were significantly higher in the AN group than in the HC group (i.e. 6.2/10 vs. 3.9/10 [*t* = 3.2, effect size  = 0.99, *P* = 0.003]); however, ratings of neutral images between groups were not different (i.e. 1.2/10 vs. 1.1/10 [*t* = 0.2, effect size  = 0.06, *P* = 0.85]; Table 1). Interestingly, as can be seen in Figure S1, no obvious increases or decreases were observed in anxiety ratings over the duration of the experiment, which might have been expected.

### Correlations between symmetry-associated anxiety ratings, OCI-R scores, and HADS scores

In the AN group, the anxiety ratings associated with viewing the symmetry images were significantly correlated with the scores obtained for ordering (rho  = 0.465, *P* = 0.04) and with the scores on the HADS (rho = 0.535, *P* = 0.019). Similarly, in the HC group, anxiety ratings were associated with viewing the symmetry images and were significantly correlated with the scores obtained for ordering (rho = 0.645, *P* = 0.001) and with scores on the HADS (rho = 0.587, *P* = 0.003). In the HC group, anxiety ratings were also correlated with obsessing scores (rho = 0.575, *P* = 0.004).

### Generic group-activation maps

The AN group showed significant activation in the bilateral parietal (x = 26, y = −66, z = 34; x = −1, y = −57, z = 52) and occipital (x = 25, y = −71, z = 13; x = 30, y = −43, z = −6) lobes in response to symmetry/order images compared to neutral images (Table 1 and Figure S2). In the HC group, a similar pattern of brain activation was seen in the parietal and occipital lobes, but activation was also seen in the right prefrontal lobe (x = 37, y = 54, z = 14) (Table 2, Figure S3).

**Table 2:** Group comparison between healthy controls and patients with AN for activation in response to symmetry/order images.

Brain region	BA	Side	Talairach's coordinates			No. of voxels	*P*
			X	Y	Z		
HC>AN							
Frontal Lobe, Superior Frontal Gyrus	10	R	22	60	−1	202	0.0002
Parietal Lobe, Precuneus	7	R	22	−67	36	99	0.0007
AN>HC							
None							

BA, Brodmann's area; Voxel-wise *P*-value <0.05 and cluster-wise *P*-value <0.002. At this level, the cumulative number of expected false-positive clusters in the group comparison was <1.

### Group Comparisons

**Figure 2:**
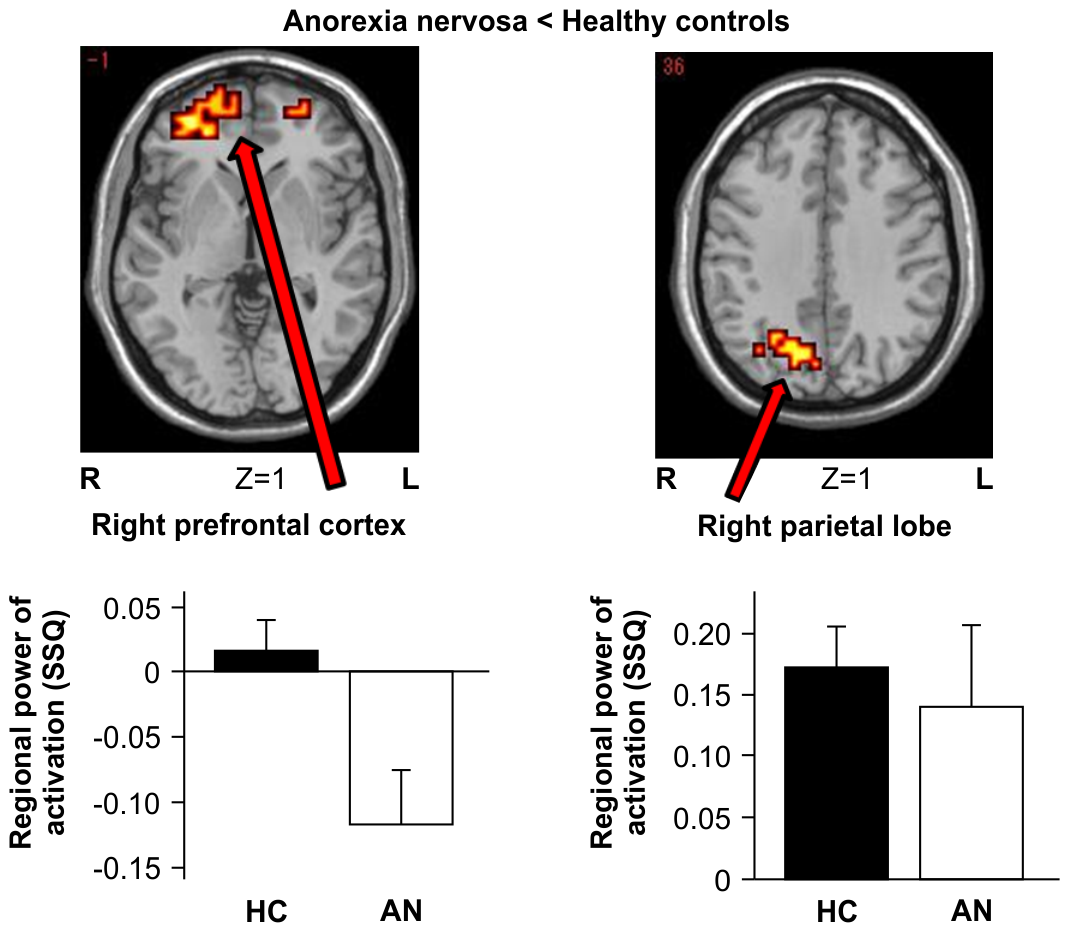
Between-group map activation showing activation provoked by symmetry-symptom images compared with neutral images in women with AN vs. healthy controls (HC). Z numbers are Talairach Z coordinates. Bright yellow indicates a large difference between groups, and dark orange indicates a small difference between groups. The colour scale of the significant clusters goes from dark red to yellow, denoting the increasing difference between the groups (yellow  =  groups most different). The bar charts show the mean power and standard error of brain activation (goodness-of-fit statistics: SSQ) extracted from the illustrated clusters separated for AN and HC.

We performed the analysis by calculating task specific activation (symmetry/order vs control) in each group at first, and then compared these task specific activations between groups. Compared with the HC group, the AN group had significantly reduced activation in the right parietal cortex (x = 22, y = −67, z = 36) (BA 7) and right prefrontal cortex (rPFC, x = 22, y = 60, z = −1) (BA 10) (Table 2, Fig 2). Moreover, no brain area showed stronger activation in the AN group relative to the HC group.

### Correlational analyses

**Figure 3:**
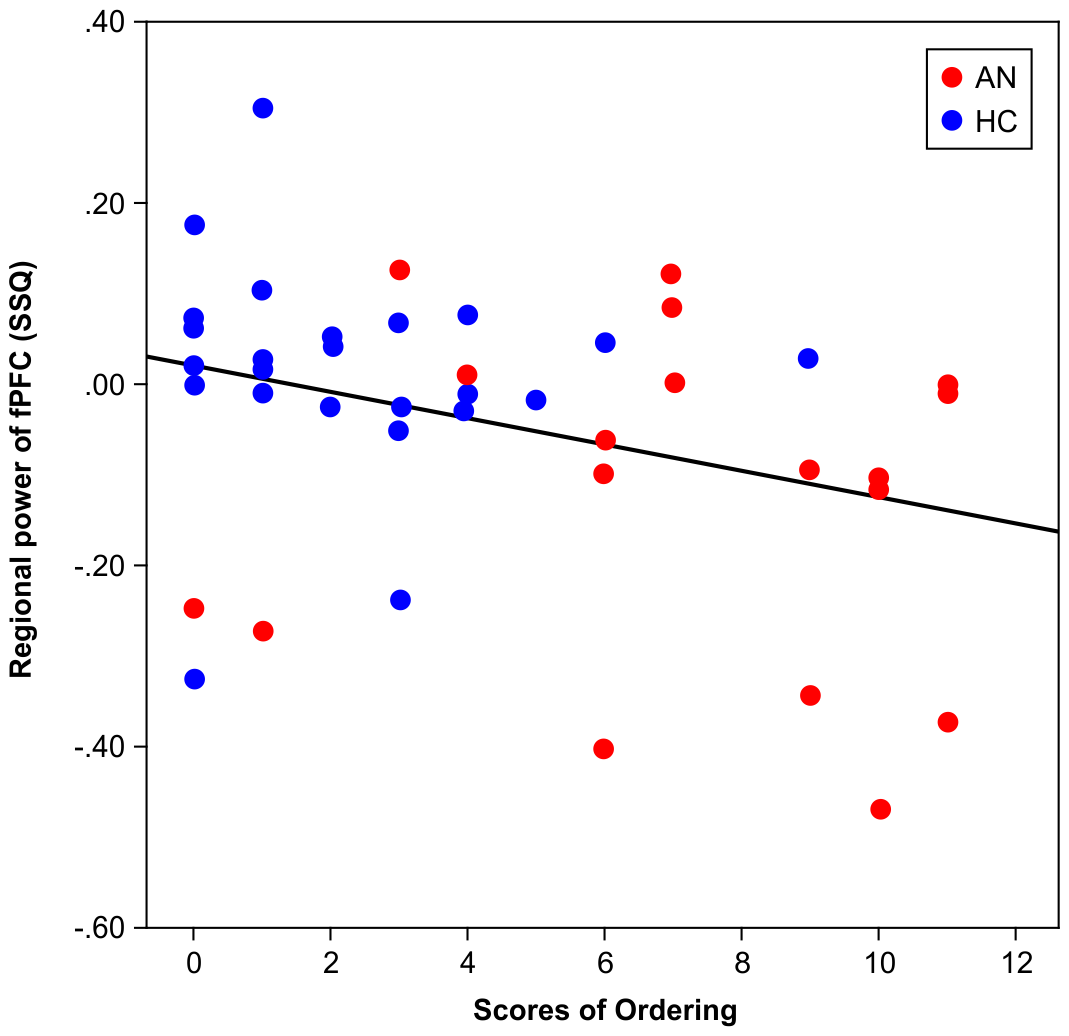
Scatter graph for rPFC activation and scores for ordering.

Participant age, duration of ED, anxiety rating for the task, OCI-R scores, and EDEQ scores, within the AN group and within the HC group, did not correlate with brain. Therefore, a multiple regression analysis was performed using the whole participant sample (AN+HC) and revealed that activation in the right PFC was inversely correlated with ordering scores (beta  = −0.325, *P* = 0.033) (Figure 3).

## Discussion

### Data summary

This fMRI study of individuals with AN and HC investigated neural responsiveness to images that provoked concern for symmetry/order. During the provocation of symmetry/order-associated symptoms, the AN group showed higher anxiety than the HC group; in both the groups, anxiety ratings correlated with how individual participants scored on the ordering sub-scale of the OCI-R and on the HADS. Generic group activation maps showed that, in the AN group, there was significant activation in the bilateral parietal and occipital lobes in response to symmetry/order images compared to neutral images. In the HC group, a similar activation pattern was seen in the parietal and occipital lobes, however, we also observed activation in the right prefrontal lobe. Group comparisons showed that during symptom provocation, the right parietal lobe and rPFC were less activated in the AN group than in the HC group. Moreover, using data from the whole group (AN+HC), activation of the rPFC during symptom provocation was inversely correlated with the severity of symmetry/ordering symptoms.

### Subjective anxiety during the performance of the symmetry/ordering tasks

As might be expected, patients with higher levels of symmetry/ordering symptoms were more anxious during symptom provocation. The anxiety ratings associated with these images were significantly higher than those associated with neutral images, indicating that the experimental manipulation was successful. However, the effect size of the subjective rating of anxiety (d = 0.98) was less than that reported using the same task in a study involving a paediatric OCD group (d = 4.58; Gilbert et al. 2009), i.e. patients diagnosed with OCD had higher scores than patients with AN.

### Brain activation during symptom provocation

Task-induced neural activations observed in the HC group were consistent with the findings of a study on healthy subjects that reported increased rCBF in the visual, parietal, and dorsal prefrontal cortices during the presentation of pictures of asymmetrically and symmetrically arranged objects [39]; this suggests that visuospatial recognition and cognitive control are both involved in the performance of such tasks.

During task performance in this study, the AN group showed reduced activation in the right parietal lobe, including the precuneus region, when compared to HCs. The reduced activity in this area of the brain is not surprising since the parietal lobe is thought to be involved in visuospatial cognition, which is a key aspect of the task employed in this study [49]. Regarding the subject of laterality, there is an inconsistency between our findings and those of a previous neuroimaging study of AN [50]. However, the abnormalities that we observed in the right hemisphere have been reported repeatedly. For example, reduced rCBF in the right parietal lobe area has been documented in resting-state studies [51], and a decrease in parietal lobe volume has been reported by structural studies (over and above a generalised reduction in brain volume) [52]. In a case study that reported structural changes over cycles of weight loss and recovery, a volume reduction in the right parietal lobe was only seen in the weight-loss condition [53]. Thus, a loss in right parietal volume might arise from starvation, but may help to subsequently maintain the disorder. The reduced activation observed in the right parietal lobe in the AN group is consistent with findings from individuals with OCD, in whom structural abnormalities in the parietal lobe are associated with the severity of ordering symptoms; scores on the symmetry/ordering dimension have been reported as being negatively correlated with grey matter volume in the parietal lobe in OCD [54].

During task performance in the present study, the AN group showed reduced dorsal PFC activation, but similar visual cortex activation, when compared to the HC group. Reduced PFC activation in individuals with AN has been reported in studies of resting-state activity [55], [56] and also in studies of symptom-provocation that employed stimuli related to social anxiety [31], food intake [25], body-checking [26], and body image distortion [20], [22]. Reduced PFC volume has also been shown in brain structural studies using MRI [15], [57]. Moreover, a review of ED symptoms and organic pathology published by [23] reported that symptoms akin to those seen in ED are associated with right frontal lobe damage. Thus, the present data showing reduced PFC activation in patients with AN in response to symmetry/ordering stimuli are consistent with the results of several studies that used other types of disorder-associated provocations. The data are also consistent with the results of a study on patients with OCD that reported an association between symmetry/ordering and functional abnormalities in the dorsal PFC based on the fact that: (a) patients with OCD had poorer set shifting abilities than controls, and b) symmetry/ordering symptoms were negatively associated with set shifting in patients [58]. In addition to the reduced rPFC activation in the AN group during task performance, we found that rPFC activation was negatively correlated with ordering scores in the whole group (AN+HC), although this negative correlation was not significant in the individual groups, possibly because of low statistical power. This negative correlation indicates that participants with high ordering scores had reduced activation in the rPFC while viewing the messy pictures, and this effect was common to both the AN and HC groups. Individuals with strong symptoms related to ordering may have less capacity to exercise top-down cognitive control when confronted with symptom-provoking images. The reason why this negative correlation was not significant in the individual groups could be due to low statistical power; it might also be a shared feature common to both AN and HC. In general, studies on cognitive emotional regulation have found that reappraisal of negative emotion activates PFC systems that support the selection and application of reappraisal strategies. The PFC also manages activity in appraisal systems, such as the amygdala or insula, in accordance with the goal of reappraisal [59]. On this basis, activation of the amygdala might have been expected in our study; however, this was not observed, and this could be due to a lack of provocation-inducing anxiety at a level high enough for the amygdala to detect.

In a study of paediatric patients with OCD [38], the performance of a task similar to what was used in the current study was associated with reduced activation in the right insula and right thalamus. The discrepancy between this previous study and the current findings could be related to the difference in participant age groups (children versus adults, respectively). It is also possible that different neural substrates underlie the response or the level of response to the symmetry/ordering task in AN and OCD. For example, the OCD/OCPD symptomatology, which is often co-morbid with AN, may have an aetiology distinct from that of OCD/OCPD in terms of brain activation.

### Limitations

This study has several limitations. First, the possibility of a medication effect cannot be ruled out, as only three participants with AN were drug naïve. Furthermore, factors such as malnutrition might have affected the patients, as their BMIs were significantly lower than those of the HCs. Another limitation is that this study used a 1.5 T MR scanner and a relatively long TR (4 s), which could have impacted the signal-to-noise ratio. Moreover, had we extended the number of experimental presentations to coincide with our present alternating block design, we might have been able to better control the novelty of the images perceived by the participants. In the symmetry/ordering task, we measured subjective anxiety but did not have any other referential condition, such as non-specific anxiety provocation. Therefore, it might be difficult to distinguish other emotional effects such as anxiety from brain activity specific to symmetry/ordering, although there was no significant correlation between brain activation and anxiety ratings. Finally, we conducted fMRI analysis over the whole-brain to identify regions associated with body checking. This was followed by correlational analyses involving the brain areas identified; this raises the risk of ‘double dipping’ – using the same dataset for selection and selective analysis.

## Conclusions

This fMRI study on individuals with AN focused on OCD/OCPD-related symptomatology, which is often co-morbid with AN [4]. Compared to HCs, participants with AN showed higher anxiety during task performance, which was correlated with their ordering scores; thus, in terms of symmetry/ordering, participants with AN exhibited similar characteristics to those individuals with OCD. Moreover, the reduced activation observed in the right parietal lobe in the AN group is consistent with findings from individuals with OCD showing that structural abnormalities in the parietal lobe are associated with the severity of ordering symptoms, i.e. scores on the symmetry/ordering dimension have been reported as being negatively correlated with grey matter volume in the parietal lobe in OCD [54]. The generic group activation maps showed that both HC and AN groups showed increased activation in the rPFC in response to symptom provocation. However, group comparisons showed lesser activation in the rPFC (and right parietal lobe) in the AN group compared to the HC group. Of note, the pattern of brain activation provoked by symmetry/ordering stimulations in patients with AN in the current study, is not consistent with the findings of OCD in a previous study [38]. These findings indicate that even though symmetry/ordering symptomatology in AN is similar to OCD at the behavioural level, AN may have an aetiology distinct from that of OCD/OCPD in terms of brain activation, especially in regions such as the rPFC. This discrepancy may further help our understanding of brain substrates that underlie AN and may also aid in the development of new therapeutic strategies.

Based on the pattern of brain activation that we observed in this study, as well as the inverse correlation we found between the levels of symptom-associated anxiety and OCI-R and HADS scores, we propose that the ability to activate the rPFC to cognitively deal with symptom-associated stimuli is diminished in individuals with AN, which results in an emotional response (e.g. anxiety). This seems to be the most parsimonious explanation of our data, and if our findings are confirmed, it is likely that the broadly-based neurobiological models of AN that propose excessive ‘top-down’ prefrontal control as a core element of AN aetiology might have to be re-appraised.

## Supporting Information

Figure S1 **Time course of anxiety ratings of each block.** AN_sym: anxiety rating for symmetry images shown to patients with AN; AN_con: anxiety rating for control images shown to patients with AN; HC_sym: anxiety rating for symmetry images shown to healthy controls; anxiety rating for control images shown to healthy controls.
(TIF)

Figure S2 **Group averaged brain activation for the symmetry/ordering task in patients with AN (n = 20), corrected to elicit less than one false positive 3D cluster for the whole map.** Eleven representative axial slices (Talairach z-coordinates: −24, −16, −8, 0, 8, 16, 24, 32, 40, 50, 60 mm) are shown in radiological convention (the right hemisphere is on the left side of the image). The colour scale of the significant clusters goes from dark red to yellow, denoting the increasing strength of the response (yellow  =  most strongly activated).
(TIF)

Figure S3 **Group averaged brain activation for the symmetry/ordering tasks in HCs (n = 24), corrected to yield less than one false positive 3D cluster for the whole map.** Eleven representative axial slices (Talairach z-coordinates: −24, −16, −8, 0, 8, 16, 24, 32, 40, 50, 60 mm) are shown in radiological convention (the right hemisphere is on the left side of the image). The colour scale of the significant clusters goes from dark red to yellow, denoting the increasing strength of the response (yellow  =  most strongly activated).
(TIF)
