# Correction: Provocation of Symmetry/Ordering Symptoms in *Anorexia nervosa*: A Functional Neuroimaging Study

**DOI:** 10.1371/journal.pone.0106295

**Published:** 2014-08-18

**Authors:** The PLOS ONE Staff

There is an error in affiliation 2 for author Samantha J. Brooks. Affiliation 2 should be: Department of Psychiatry and Mental Health, University of Cape Town, Cape Town, South Africa.
