# Supplementary figures and images for: Provocation of Symmetry/Ordering Symptoms in Anorexia nervosa: A Functional Neuroimaging Study
Source: PLoS One. 2014 May 20;9(5):e97998. doi: 10.1371/journal.pone.0097998 (PMC4028263; doi:10.1371/journal.pone.0097998)

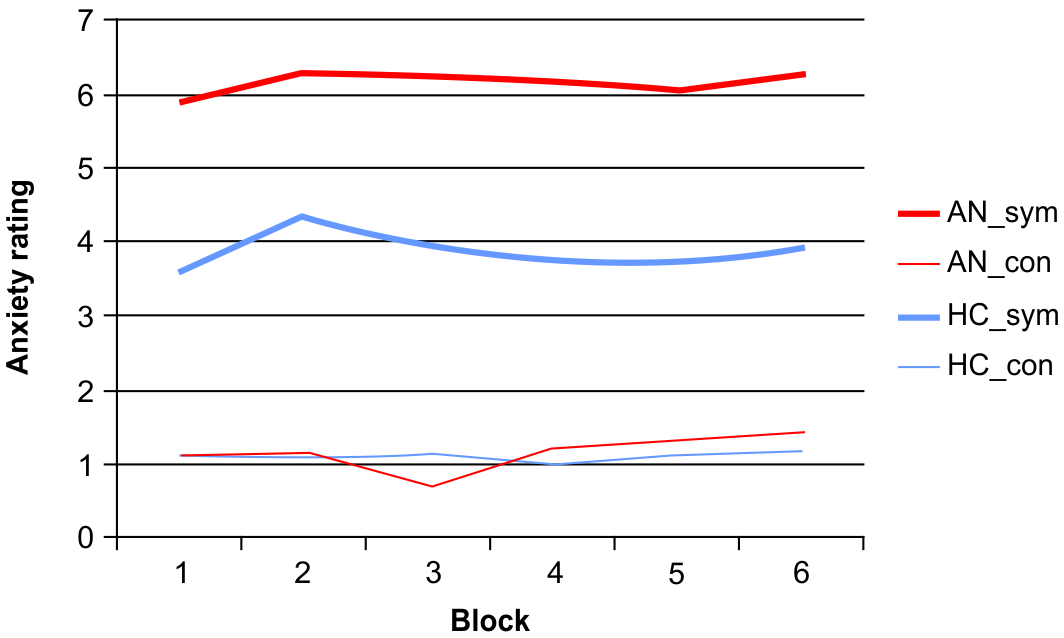

Supplement: Figure S1 — Time course of anxiety ratings of each block. AN_sym: anxiety rating for symmetry images shown to patients with AN; AN_con: anxiety rating for control images shown to patients with AN; HC_sym: anxiety rating for symmetry images shown to healthy controls; anxiety rating for control images shown to healthy controls. (TIF) [file pone.0097998.s001.tif]

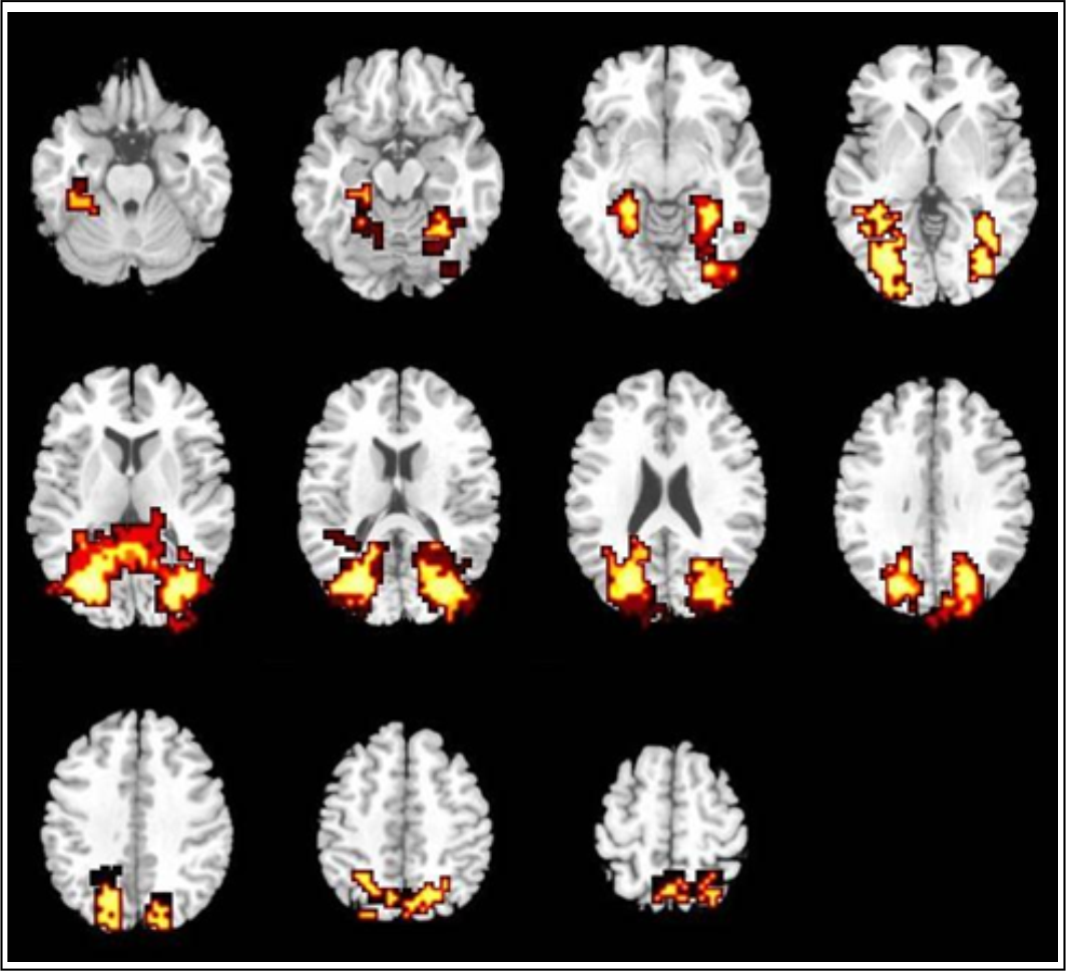

Supplement: Figure S2 — Group averaged brain activation for the symmetry/ordering task in patients with AN (n = 20), corrected to elicit less than one false positive 3D cluster for the whole map. Eleven representative axial slices (Talairach z-coordinates: −24, −16, −8, 0, 8, 16, 24, 32, 40, 50, 60 mm) are shown in radiological convention (the right hemisphere is on the left side of the image). The colour scale of the significant clusters goes from dark red to yellow, denoting the increasing strength of the response (yellow = most strongly activated). (TIF) [file pone.0097998.s002.tif]

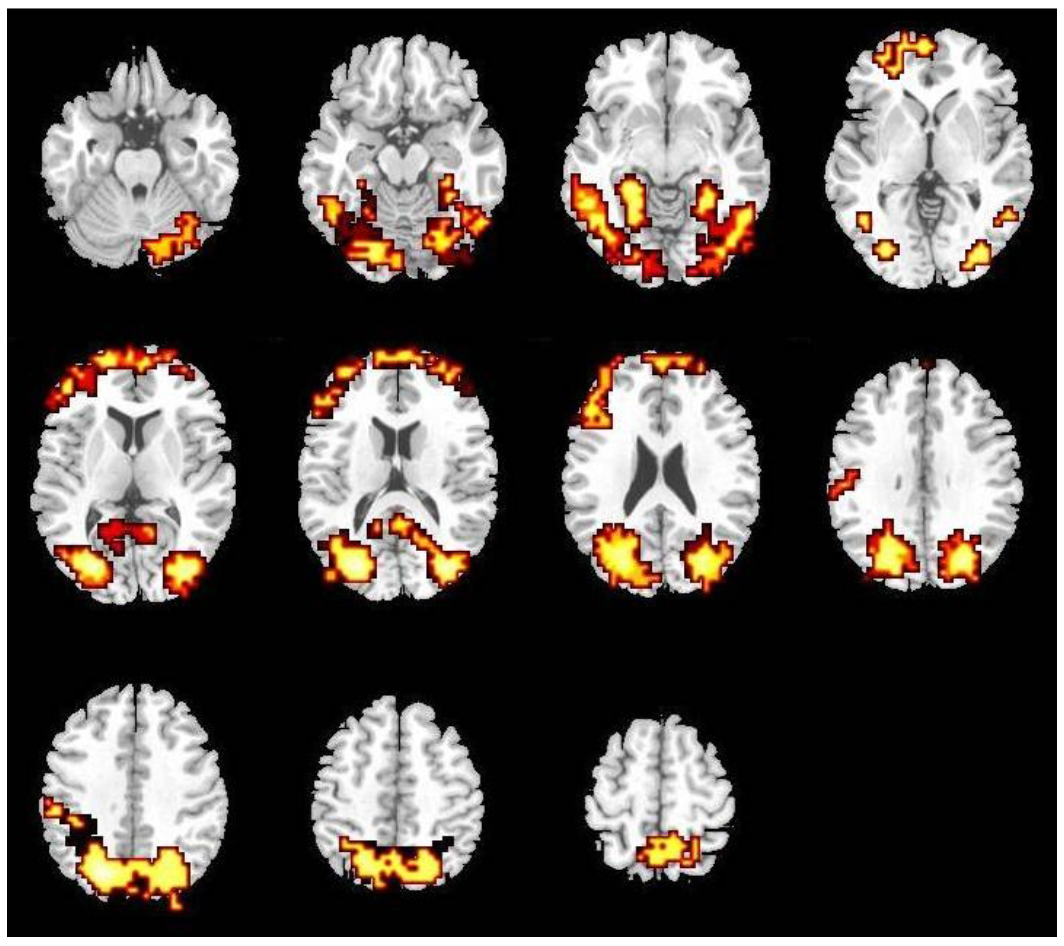

Supplement: Figure S3 — Group averaged brain activation for the symmetry/ordering tasks in HCs (n = 24), corrected to yield less than one false positive 3D cluster for the whole map. Eleven representative axial slices (Talairach z-coordinates: −24, −16, −8, 0, 8, 16, 24, 32, 40, 50, 60 mm) are shown in radiological convention (the right hemisphere is on the left side of the image). The colour scale of the significant clusters goes from dark red to yellow, denoting the increasing strength of the response (yellow = most strongly activated). (TIF) [file pone.0097998.s003.tif]
